# Evaluation of efficacy and safety of aspirin combination treatment in treating patients with coronary heart disease: A protocol for systematic review and meta-analysis

**DOI:** 10.1097/MD.0000000000028848

**Published:** 2022-02-18

**Authors:** Xueling Wang, Lin Han, Jiyuan Yang

**Affiliations:** a Department of Cardiovascular Medicine, Beijing Hospital of Integrated Traditional Chinese and Western Medicine, Beijing, China; b Department of Cardiology, Beijing Luhe Hospital, Capital Medical University, Beijing, China.

**Keywords:** aspirin, coronary heart disease, efficacy, meta, treatment

## Abstract

**Background:**

Coronary heart disease (CHD) has a high incidence rate as a cardiovascular condition, primarily affecting the elderly and middle-aged individuals. CHD has debilitating effects on the standard of life of the elderly, and affecting their physical and psychological health. Reportedly, using aspirin alone is less effective as a first line of treatment for CHD. Therefore, this systematic review and meta-analysis will synthesize evidence on the effectiveness and safeness of aspirin combination treatment in treating patients with CHD.

**Methods:**

A comprehensive meta-analysis is to be performed to evaluate the effectiveness and safety of aspirin combination treatment for CHD patients. A search will be performed on PubMed, EMBASE, Cochrane Central, WanFang, and Chinese National Knowledge Infrastructure till December 25, 2021 to identify randomized controlled trials, assess all related studies on the aspirin combination treatment in treating patients with CHD. In this systematic review, we will adopt the second version of Cochrane risk of bias assessment tool to assess the bias risk in all studies that fulfil the eligibility conditions. Two authors will separately conduct the study selection process, risk of bias assessment, and data extraction. Moreover, a random-effects meta-analysis will be conducted to synthesize evidence for all outcomes. Provided there is sufficient homogeneity among the studies, we will perform meta-analysis. *I* ^2^ test will be employed to assess the heterogeneity of the outcomes.

**OSF registration number:**

10.17605/OSF.IO/MDTCA

## 1 Introduction

Despite monumental advancements in intensive medical therapy that have enabled to reduce fatalities associated with heart conditions, coronary heart disease (CHD) remains a primary cause of mortality globally, primarily affecting the elderly and middle-aged individuals. In 2017, the global mortality related to CHD was 116.9 for every 100,000 of the population.^[^1^,^^2^^]^ In China, CHD related fatalities reached 124 per 100,000 individuals in 2017, which was a 20.6% increment relative to CHD related deaths in 1990.^[3]^ CHD is characterized by repeated relapse, acuteness, and severe clinical manifestations, if left untreated, CHD may induce myocardial infarction, leading to sudden cardiac arrest. CHD is a life-threatening condition whose pathogenesis is linked to hemadostenosis and coronary atherosclerosis drastically affecting the standard of life of patients.^[2]^ Improving myocardial blood supply is critical as a clinical treatment, which typically uses antiplatelet agents. Considering the adverse outcomes associated with CHD, better treatment methods are urgently needed to improve the prognosis of patients with CHD.

Aspirin is classified as a non-steroidal anti-inflammatory drug, and acetylsalicylic acid is its principal component.^[4]^ Aspirin is commonly administered to treat numerous types of diseases and conditions, which includes relieving pain and fever, myocardial infarction, inflammation, cardiovascular morbidity, and some forms of cancer.^[^4^,^^5^^]^ As a chronic inflammatory disease, CHD presents inflammation throughout each stage of atherosclerosis, and it is likely that inflammation is a common link or pathway in the pathogenic functionality of several atherogenic factors.^[^6^,^^7^^]^ Clinically, aspirin has had limiting effects on the expression of inflammatory cytokines, antiplatelet, and antiatherosclerosis, preventing thrombosis.^[8]^ As an anticoagulant, the efficacy of aspirin has been comprehensively affirmed in clinical settings. Besides, aspirin is relatively low priced, so it has become a mainstay drug to treat CHD. However, recently, there has been increasing reports that the long-term use of antiplatelet agents can lead to adverse reactions. Thus, it is imperative to enhance the anticoagulant effect of aspirin by combining with other drugs. In this meta-analysis, we will assess the efficacy and safeness of combining aspirin to treat individuals with CHD.

## 2 Methods

### 2.1 Protocol registration and reporting

The development of this protocol will follow the Preferred Reporting Items for Systematic Review and Meta-Analysis Protocol (PRISMA-P) guidelines.^[9]^ The PRISMA-P checklist will be employed as an indicator to demonstrate adherence to the guidelines. This protocol is registered at the Open Science Framework (OSF) (registration number 10.17605/OSF.IO/MDTCA).

### 2.2 Information source

A comprehensive meta-analysis is to be performed to evaluate the effectiveness and safety of aspirin combination treatment for CHD patients. A search will be performed on PubMed, EMBASE, Cochrane Central, WanFang, and Chinese National Knowledge Infrastructure till December 25, 2021 to identify randomized controlled trials, assess all related studies on the aspirin combination treatment in treating patients with CHD. Moreover, we will perform another comprehensive search in Google Scholar to retrieve grey literature. Additionally, we will manually search all the reference lists in retrieved studies to identify other related articles. The following keywords will be used to perform the search:

1. “aspirin,”
2. “coronary heart disease,” and
3. “clinical effect.”

The language will be restricted to Chinese and English.

### 2.3 Criteria for considering studies for this review

#### 2.3.1 Types of studies

All randomized controlled trials that evaluate the effectiveness and safety of aspirin combination treatment in treating patients with CHD will be included.

#### 2.3.2 Types of participants

Participants include all patients with CHD that have been diagnosed according to the CHD diagnosis criteria of the clinical diagnosis standards established by the WHO, irrespective of gender and nationality, with normal renal and hepatic functions, who had not taken any antiplatelet agents or anticoagulants recently.^[10]^

#### 2.3.3 Types of intervention

The experimental cohort was administered aspirin in combination with other drugs, irrespective of dosage, meanwhile the control was only administered aspirin, other treatment, or no intervention; however, patients could intake non-anticoagulants and non-lipid-lowing medication.

#### 2.3.4 Outcome measures

Outcome measures included all-cause mortality, vascular deaths, response rate, incidence of adverse reactions, direct- and indirect-tests of disease severity, cerebrovascular events, and non-fatal events.

### 2.4 Data collection and analysis

#### 2.4.1 Selection of studies

A pair of authors will separately perform a screening of titles/abstracts of all potential articles and code them as either “retrieve” (eligible or potentially eligible/unclear) or “do not retrieve.” The authors will gain access to comprehensive texts and study reports of each eligible article and a pair of authors will autonomously screen them for inclusion, and record exclusion reasons during the process. All disagreements shall be mediated via discussion or by consulting another independent author. All duplicated reports and collate multiple reports of the same study will be identified and omitted. Accordingly, each study, rather than each report, will be the unit of interest in the review.

#### 2.4.2 Data extraction and management

The two authors will independently will extract data from the original publications and tabulate the obtained information in Excel spreadsheets, including the author's first name, sample size, publication date, mean gender and age of participants, interventions in the experimental and control groups, treatment duration, and prognostic indexes. Besides presenting data for analytical purposes, the extracted data will comprise descriptive study characteristics.

#### 2.4.3 Assessment of risk of bias in included studies

We will adhere to the quality assessment norms suggested in Cochrane handbook when evaluating the quality of the included research. The assessment items encompass random allocation (selection bias), allocation concealment (selection bias), and blinding (performance bias). The pair of authors will operate autonomously in resolving any issues in dispute through discussion, and outstanding differences shall be mediated with the aid of another author (third).

#### 2.4.4 Measures of treatment effect

To represent dichotomous outcomes, we will adopt the risk ratio with its 95% confidence interval as a measure of therapeutic effect. We will analyze continuous scales of measurements as mean difference with a 95% confidence interval.

#### 2.4.5 Dealing with missing data

We will extract the data that we required for this review from the primary studies. We will use the sample size that investigators indicated as randomized and extract the number of events reported in the results.

#### 2.4.6 Assessment of heterogeneity

The heterogeneity among studies shall be quantified using the *I* ^2^ statistic, where *I* ^2^ greater than 75% will be regarded as significant heterogeneity. Specifically, when *I* ^2^ > 75%, results from each article will be reported descriptively instead of combining through meta-analysis.

#### 2.4.7 Assessment of reporting biases

If the present analysis includes in excess of 10 studies, we a funnel plot of effect estimates against their standard errors will be created to explore possible small study and publication biases.

#### 2.4.8 Sensitivity analysis

If sufficient studies could be identified, we will intend to repeat the analyses without including studies with high risk of bias.

## 3 Discussion

From a clinical perspective, lifetime uptake of aspirin exhibits decent efficacy in reducing cardiovascular disease–related fatalities, strokes, and myocardial infarction.^[11]^ As a conventional antiplatelet aggregation and antithrombotic drug, Aspirin has been commonly administered to clinically treat CHD. However, solely administering aspirin is linked with several side effects, such as gastrointestinal hemorrhage and renal toxicity.^[^4^,^^8^^]^ Admittedly, past work has confirmed the efficacy of aspirin interventions. However, there is a general lack of determination of the most effective therapeutic aspirin combination treatment in treating patients with CHD. Hence, the present online meta-analysis will present an all-inclusive summary of the most recent evidence, with an emphasis on existing aspirin combination treatments. The authors hope that the findings of this study will be of practical value to clinicians, patients, and healthcare policymakers, to adopt the best treatment methods for CHD.

## Author contributions

**Conceptualization:** Xueling Wang, Lin Han, Jiyuan Yang.

**Data curation:** Xueling Wang, Lin Han, Jiyuan Yang.

**Formal analysis:** Xueling Wang, Lin Han, Jiyuan Yang.

**Funding acquisition:** Xueling Wang.

**Investigation:** Xueling Wang, Lin Han, Jiyuan Yang.

**Methodology:** Xueling Wang.

**Project administration:** Lin Han, Jiyuan Yang.

**Resources:** Xueling Wang.

**Software:** Xueling Wang, Lin Han, Jiyuan Yang.

**Validation:** Xueling Wang, Lin Han, Jiyuan Yang.

**Visualization:** Xueling Wang, Lin Han.

**Writing – original draft:** Xueling Wang, Lin Han.

**Writing – review & editing:** Xueling Wang.

## References

[1] Global, regional, and national disability-adjusted life-years (DALYs) for 359 diseases and injuries and healthy life expectancy (HALE) for 195 countries and territories, 1990–2017: a systematic analysis for the Global Burden of Disease Study 2017. Lancet 2018;392:1859–922. 30415748 10.1016/S0140-6736(18)32335-3 PMC6252083

[2] Roth GA Johnson C Abajobir A. Global, regional, and national burden of cardiovascular diseases for 10 causes, 1990 to 2015. J Am Coll Cardiol 2017;70:01–25. 10.1016/j.jacc.2017.04.052 PMC5491406 28527533

[3] Zhou M Wang H Zeng X. Mortality, morbidity, and risk factors in China and its provinces, 1990–2017: a systematic analysis for the Global Burden of Disease Study 2017. Lancet 2019;394:1145–58. 31248666 10.1016/S0140-6736(19)30427-1 PMC6891889

[4] Patrono C. Aspirin as an antiplatelet drug. N Engl J Med 1994;330:1287–94. 8145785 10.1056/NEJM199405053301808

[5] Cuzick J Otto F Baron JA. Aspirin and non-steroidal anti-inflammatory drugs for cancer prevention: an international consensus statement. Lancet Oncol 2009;10:501–7. 19410194 10.1016/S1470-2045(09)70035-X

[6] Moriya J. Critical roles of inflammation in atherosclerosis. J Cardiol 2019;73:22–7. 29907363 10.1016/j.jjcc.2018.05.010

[7] Pedro-Botet J Climent E Benaiges D. Atherosclerosis and inflammation. New therapeutic approaches. Med Clin (Barc) 2020;155:256–62. 32571617 10.1016/j.medcli.2020.04.024

[8] Hybiak J Broniarek I Kiryczyński G. Aspirin and its pleiotropic application. Eur J Pharmacol 2020;866: 172762. 10.1016/j.ejphar.2019.172762 31669590

[9] Moher D Shamseer L Clarke M. Preferred reporting items for systematic review and meta-analysis protocols (PRISMA-P) 2015 statement. Syst Rev 2015;4:01. 10.1186/2046-4053-4-1 PMC4320440 25554246

[10] Knuuti J Wijns W Saraste A. 2019 ESC Guidelines for the diagnosis and management of chronic coronary syndromes. Eur Heart J 2020;41:407–77. 31504439 10.1093/eurheartj/ehz425

[11] Grines CL Bonow RO Casey DE Jr. Prevention of premature discontinuation of dual antiplatelet therapy in patients with coronary artery stents: a science advisory from the American Heart Association, American College of Cardiology, Society for Cardiovascular Angiography and Interventions, American College of Surgeons, and American Dental Association, with representation from the American College of Physicians. J Am Coll Cardiol 2007;49:734–9. 17291948 10.1016/j.jacc.2007.01.003

